# AI Competency: Current State and Challenges

**DOI:** 10.2196/86686

**Published:** 2026-03-03

**Authors:** Sian Tsuei

**Affiliations:** 1 Department of Family Practice, University of British Columbia , David Strangway Bldg, 5950 University Blvd 3rd Floor, Vancouver, BC, V6T 1Z3, Canada, + 1 604-822-5431; 2 Department of Global Health and Population, T.H. Chan School of Public Health, Harvard University, 677 Huntington Avenue, Boston, MA, 02115, United States, 1 778-871-8292

**Keywords:** artificial intelligence, medical education, AI

## Abstract

As artificial intelligence (AI) develops, the medical education community has begun defining the relevant forms of competency. Many experts emphasize the importance of optimizing AI tools’ output or understanding the relevant technical and normative considerations around using AI tools. A recent publication in this journal showed that optimizing instructions for large language models may yield diminishing returns as such tools improve. This suggests the need for a new competency—one that focuses on choosing the appropriate AI tools. I briefly summarize the current competency domains and examples to contextualize the current state of AI competency development, highlighting the need for further synthesis. I then introduce a hierarchical framework of competencies that might assist with priority-setting around subsequent competency development work. It consists of cognitive, operational, and meta-AI domains, which respectively correspond with the knowledge around understanding, using, and choosing AI tools. The final section describes the potential challenges associated with the development of AI competency. These include traditional concerns around competency-based medical education: deciding whether and which competencies are meaningful for measuring the targets of interest; adjusting the relevant measurements to reflect the necessary temporal and institutional context; and setting up the relevant organizational support to encourage measurement of competency. This section also discusses the challenges of developing the relevant performance indicators for AI tools across different clinical contexts. Such indicators will be necessary for guiding the choice of AI tools for the clinical context, but medical educators may not have the skills to develop them. In addition to identifying potential sources for relevant indicators, the medical education community may shape physicians’ norms of practice to drive the AI industry into producing the relevant indicators. The potential for physicians to incur higher medical liability from poor choice of AI may lead them to demand more nuanced performance indicators from AI suppliers. Physicians are also in a position to do so, since the competitive AI market may provide them more bargaining power.

## Background

Given the increasing popularity and capability of modern artificial intelligence (AI) tools [1], helping students develop AI competency is becoming increasingly crucial. Although recommendations vary, the core skills generally focus on helping trainees understand the various AI tools’ technological approaches, uses, and risks, as well as the relevant ethical, social, cultural, and legal context around AI tool use [2-6].

The work from Hsieh et al [7] suggests the need for a potentially novel type of AI competency—one that focuses on the choice of AI tool. Their article showed that even though providing better instructions for large language models (LLMs) can improve their performance, the margin of improvement diminished for more advanced LLMs. This suggests that as LLMs improve, optimizing the prompt may become less important. Instead, choosing an appropriate LLM may become more critical. This implication calls into question the extent to which current medical education literature has considered the choice of LLM as a key competency.

To help medical educators and trainees critically engage with the future development of AI-related competencies, this editorial article therefore aims to (1) illustrate the state of AI competency development so that (2) it can consider worthwhile directions for this field. In what follows, I will briefly describe the current stage of AI competency development, drawing on up-to-date literature, followed by the introduction of a new taxonomy framework that can help priority-setting around future competency development work. I follow with a brief reflection on the challenges of AI competency development, first summarizing from literature the traditional concerns affecting competency development, and propose additional considerations that might uniquely affect competencies around the choice of LLM.

## Domains of AI Competencies Considered

Typically, the development of medical education competencies relies on four steps: “listing competencies, devising training programmes to fit these, utilising appropriate assessment methods and determining pass levels” [8]. Much of the current discourse around AI competence has focused on the first step, seeming to largely focus on the following domains: (1) AI fundamentals, that is, understanding the mechanics underpinning AI tool performance; (2) ethical and legal considerations, understanding the ethical and legal implications of using AI tools; (3) data analysis and management, understanding how data drives AI tools’ function and appropriate data handling practices; (4) evaluation of AI tools, assessing AI tools’ performance to ensure that they meet necessary standards; and (5) use of AI tools, understanding whether, why, when, and how to use AI tools effectively for clinical care [3,9-13]. Currently, the American Association of Medical Colleges is synthesizing AI competencies [14], and other countries’ medical associations may also wish to undertake their own adaptations to ensure local appropriateness.

Beyond competency description, some medical educators are already developing training programs and evaluation methods [15,16]. Further systematic and critical synthesis of the current AI curricular directions, evaluation methods, and impacts would be helpful for guiding subsequent efforts. Ideally, this synthesis would be able to benchmark AI competencies against established competency frameworks.

As medical educators progress along the AI-related competency-based medical education (CBME) journey, identifying which types of competencies deserve priority may be helpful. Some of the competencies may be more foundational, while others are more advanced. Table 1 describes a taxonomy that synthesizes the range of competency domains, arranging them from foundational to the most advanced.

**Table 1.** Hierarchical progression of artificial intelligence (AI) and large language model (LLM) competency.

Level of competency	Type of competency	Competency domains implicated	Goals of competency for trainees	Examples of trainee competencies
Foundational	Cognitive competency	AI fundamentals	Understand the theoretical and operational approaches, benefits, and limitations of LLMs	Explain how deep learning works
Advanced	Operational competency	Ethical and legal considerations Data analysis and management Use of AI tools	Use LLMs appropriately, recognizing ethical, social, cultural, and legal implications and limitations	Understand best practices for data storage
Most advanced	Meta-AI competency	Evaluation of AI tools Use of AI tools	Choose appropriate LLM for the clinical context	Understand relevant indicators of LLM performance

The first level—cognitive competency—helps individuals understand the development and operations of LLMs. Medical educators might illustrate the technical intuitions behind deep learning, modeling approaches, and data transformation. This can then help trainees understand why LLMs might suffer from technical limitations (eg, hallucinations, sycophancy bias, and algorithmic biases) [3,11-13].

The second level—operational competency—helps trainees use LLMs more effectively. Medical educators may teach trainees to identify the relevant clinical features of a case and refine the prompt and context to optimize the usefulness of an LLM’s response [3,12,13]. Educators may also help trainees appreciate the relevant ethical, social, cultural, and legal requirements so that data are ethically processed.

The third level—meta-AI competency—ensures that trainees can pick the most appropriate AI models. The trainees will need to identify the clinical problem, discern the markers that show that an AI model is context-appropriate, and select a suitable LLM [12]. This requires that the trainees first understand how LLMs work and how to use them, so that they can meaningfully identify the best choice of LLM for the target scenarios. Of note, only two of the competencies appear to touch on this level of competency (Table 1’s “Use of AI tools” and “Evaluation of AI tools” domains). Further development of the competencies at this level may be necessary.

## Challenges Related to the Development of AI Competency

The development of AI competency may face multiple challenges. Some of them pertain to CBME in general, and some are uniquely related to aspects of AI competency. In what follows, I will first highlight the more prominent and recent critiques of the general topic of CBME, guided by the timely and thorough review of Hamza et al [17]. I connect these concerns with AI competency where possible. I will subsequently consider the unique challenges of meta-AI competency. Its development may draw in the AI development industry, transcending the usual considerations related to medical education competency development.

## First Type of Challenge: General Critique of CBME

**Figure 1.**
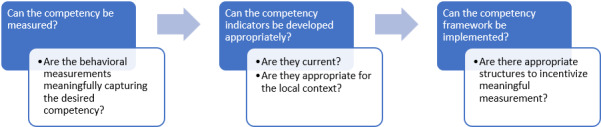
Conventional concerns of CBME.

Figure 1 summarizes the major veins of critiques of CBME. They largely question (1) whether the relevant competency can be meaningfully measured; (2) assuming that such measurement is possible, whether the measurement can be appropriately adapted; and (3) assuming that adapting the measurement is possible, whether the appropriate measurements can be practically implemented.

First, the critics challenge the appropriateness of CBME as a paradigm. Fundamentally, CBME relies on assessing observable indicators, but sometimes the target competency cannot be measured. As a result, the assessment may target “things that may be more easily measured, instead of asking the more difficult questions” [8]. This risks distracting learners from meaningfully understanding the content or reflecting on the nuances of the situation [18], which may be necessary for doctors to reason from first principles and adapt to circumstances beyond the original medical training content. For example, if trainees fail to understand that AI models can continuously evolve based on live use data, they may fail to grasp the potential for the performance of the models to drift over time. If they continue to rely on AI tools to the same extent even as the tools’ performance drops, this can undermine care quality. As the medical education community proposes and synthesizes AI competency, carefully considering which competencies are truly meaningful representations of the underlying cognitive, attitudinal, and behavioral targets will be key.

Second, critics are concerned that competency measurements may not be adequately adjusted so that the measurement actually captures the relevant competency. From a temporal standpoint, competencies need to be regularly updated to keep up with the times [19]. This is especially relevant for AI competencies. AI tools are developing rapidly, and the way they are used may drastically change in a few years. What are the indicators that should trigger the necessary updates? Should competencies simply be reviewed at a prespecified interval? These are key questions for AI-related CBME. Furthermore, from an institutional standpoint, different health care systems may have unique cultures, workflows, and resources, requiring local adaptation [20,21]. Although the competencies may be stable, the demonstration—and measurement—of those competencies may need to be adapted to the context [17,19,22]. In the context of AI, local resource constraints, population demographics, and patient preferences may further complicate these decisions, highlighting the importance for medical educators of developing local expertise regarding AI and health systems so that the measurement of the competencies can be adapted appropriately.

Third, organizational structures may not provide appropriate incentives for teachers and learners to check competence [17]. This can be problematic if modern AI accelerates the pace of service delivery and the health care system fails to concomitantly introduce appropriate incentives to encourage in-depth assessment and feedback. Health system and change management expertise are necessary complements for meaningful AI-related CBME.

## Second Type of Challenge: AI Governance Challenges Undermining Meta-AI Competency Development

Aside from these conventional critiques of competency, developing meta-AI competency may face unique challenges. If physicians are to choose AI tools for specific contexts, they need to know how the AI tool performs within various clinical contexts (eg, different populations, clinical specialties, and care providers). Without such performance indicators, choosing the AI tools for the relevant patient encounters may be challenging. These competencies are therefore predicated on the presence of accurate and meaningful performance indicators of various AI tools in a range of contexts.

Meta-AI competency may be challenging for medical educators to develop. Whereas conventional medical competencies can draw directly from medical knowledge, AI performance indicators require deep AI expertise, which may be outside current medical educators’ skill sets. Depending on the AI industry to provide useful performance metrics requires at least some reliance on effective governance of the AI industry.

Approaches to such governance seem to be emerging along a spectrum. The European Union (EU) represents the more stringent end: Article 10, Paragraph 3 of the EU AI Act specifies that “[AI] providers must ensure that the training, validation, and testing datasets shall be relevant, sufficiently representative, and, to the best extent possible, free of errors and complete according to the intended purpose.” The implication is that if an AI tool is intended for use in dermatology, for example, but its dataset only contains images from a specific demographic or body part, a developer would need to document this limitation to comply with the data governance requirements. The “intended purpose” must clearly define the scope, which can include medical specialties and specific contexts of use.

On the other end of the spectrum, the United States seems to be much friendlier to the AI industry, focusing on the economic opportunities associated with AI proliferation and providing minimal effective guardrails around AI use. In fact, even though the Food and Drug Administration (FDA) is empowered to regulate AI tools, regulation is severely lacking, and entire columns are sometimes missing in the FDA database [23]. AI tools may therefore have little incentive to provide meaningful evidence of their performance in different contexts, which would undermine the development of relevant meta-AI competency.

Implicitly, each approach reflects the value society places on stringent regulation of an emerging technology and on safeguarding patients’ health. For medical educators, however, what might matter more is considering how teachers and learners ought to adapt when the relevant governance approaches fail to drive the disclosure of necessary AI performance indicators.

Educators may highlight to students the possibility of identifying the necessary information elsewhere. Performance indicators from the EU AI Act’s database might be transferable to the population of interest, because the AI supplier validated the AI tool using a population of similar demographic composition. Such adaptation should note, however, that different health care systems deviate from each other in various ways [24], so careful consideration of whether to use the tool and how to do so appropriately will be key.

Even more critically, the medical education community can use their educational power to drive market change. Rather than awaiting regulatory saviors, educators can step in to drive the trajectory of competency assessment. Medical educators have the power to institutionalize behavioral norms among medical trainees. Medical training is rigorous and formalized so that the trainees graduate as a group of individuals who are homogeneous in skills, aptitudes, and values [25]. If medical trainees learn to consistently review relevant performance indicators before using AI tools, this behavioral norm can be a meaningful bargaining force in the AI industry, potentially pushing AI suppliers to change the relevant information for their AI products. AI suppliers may realize that they can capture more physicians as customers if they provide more indicators of their tools’ performance. Essentially, medical educators’ power to influence curricula can trickle down and affect doctors’ behavioral norms, which can then change industry norms.

However, critics may argue that past efforts from medical education failed to influence industry standards. A good example is medical education’s influence on the medical documentation process and electronic medical record (EMR) industry. First, despite medical educators’ efforts to teach proper charting, medical doctors’ charting standards are still problematic [26]. This suggests that practicing doctors may shed their training if it goes against their style of practice. If so, doctors may never end up pushing the industry to provide relevant metrics, because they might not care about academic lessons during training. The attending physician might reach for the LLM most commonly used without vetting its metrics for the specific context.

Second, even if physicians’ practices change and they push against the industry, there is no promise that the vendors will make the needed changes. For example, physicians’ concerns over many EMR platforms’ poor usability have been well documented since 2004 [27], but complaints about this have persisted into 2025 [28,29].

The unique context of the current medical AI industry may refute these concerns. First, the potential for legal risks related to using medical AI tools for clinical services may drive physicians to more closely adhere to training recommendations in carefully vetting AI tools. Administrative capabilities aside, AI tools’ capacity to support medical diagnoses can seriously alter the course of treatment for patients. Improper vetting of AI tools can significantly increase physicians’ liability [30], suggesting that physicians may have much interest in adhering to training recommendations for AI tool vetting.

Second, the current AI market structure appears to provide buyers with more power relative to suppliers, suggesting that AI vendors may wish to cater to doctors’ concerns. Foundational LLMs are becoming substitutable commodities [31], as many LLMs provide similar levels of performance. Furthermore, these foundational models help lower the entry cost for secondary AI tool developers to release similar AI tools that compete intensely for consumers. The boom in AI scribes is one such example, and aggressive attempts to improve physicians’ user experiences by offering ways to integrate their products into different EMR platforms is an unsurprising consequence.

## Conclusion

Medical education experts have proposed several domains and competencies related to use of AI, and further synthesis of such work can be beneficial. I proposed a hierarchical framework of AI competency that can help medical educators discern the order of priority for competency development. The meta-AI competency, which is at the top of the hierarchy, may be particularly challenging to measure and implement. In addition to conventional challenges related to CBME, medical educators require effective AI governance to identify AI performance indicators that would most support trainees in selecting appropriate AI tools for the clinical context. Given the current market structure, the community of medical educators may have an opportunity to leverage their power in influencing the norms of practice to drive further AI performance indicators.

## Abbreviations

AI: artificial intelligence
CBME: competency-based medical education
EMR: electronic medical record
EU: European Union
FDA: Food and Drug Administration
LLM: large language model
